# Speed Control for Leader-Follower Robot Formation Using Fuzzy System and Supervised Machine Learning

**DOI:** 10.3390/s21103433

**Published:** 2021-05-14

**Authors:** Mohammad Samadi Gharajeh, Hossein B. Jond

**Affiliations:** 1 Polytechnic Institute of Porto, 4200-465 Porto, Portugal; mmasa@isep.ipp.pt; 2 Department of Computer Science, VSB–Technical University of Ostrava, 17, Listopadu 2172/15, 708 00 Ostrava, Czech Republic

**Keywords:** autonomous robot, speed control, intelligent technique, fuzzy system, supervised machine learning

## Abstract

Mobile robots are endeavoring toward full autonomy. To that end, wheeled mobile robots have to function under non-holonomic constraints and uncertainty derived by feedback sensors and/or internal dynamics. Speed control is one of the main and challenging objectives in the endeavor for efficient autonomous collision-free navigation. This paper proposes an intelligent technique for speed control of a wheeled mobile robot using a combination of fuzzy logic and supervised machine learning (SML). The technique is appropriate for flexible leader-follower formation control on straight paths where a follower robot maintains a safely varying distance from a leader robot. A fuzzy controller specifies the ultimate distance of the follower to the leader using the measurements obtained from two ultrasonic sensors. An SML algorithm estimates a proper speed for the follower based on the ultimate distance. Simulations demonstrated that the proposed technique appropriately adjusts the follower robot’s speed to maintain a flexible formation with the leader robot.

## 1. Introduction

With increasing computing power, fast progress in sensor and actuator design, and low-cost production, robotic systems have been in ever-increasing demand and application. The ultimate goal of robotic engineers and researchers is to achieve fully autonomous navigation in indoor/outdoor environments [1]. The control system of an autonomous wheeled mobile robot perceives its environment via embedded sensors and controls the robot’s navigation. An effective control algorithm navigates the robot through a (near-) optimal collision-free path from a start position to the target [2,3]. The path’s optimality is measured with respect to the traversed path length and navigation time, etc. Navigation in unknown environments with stationary and/or mobile obstacles is the main challenge for wheeled mobile robots [4,5].

Efficient speed control is essential to the navigation and path tracking of wheeled mobile robots. Employing intelligent and knowledge-based controllers for adjusting the robot speed autonomously during navigation has been addressed in the literature. Kodagoda et al. [6] have developed and implemented fuzzy controllers for the steering and speed control of an autonomous guided vehicle. They applied fuzzy logic for steering control and with the suitable incorporation of a braking controller, the stability of the vehicle is guaranteed. Dursun and Durdu [7] have presented a method for the speed control of a DC motor utilized in robots and countless industrial applications based on sliding mode control and analysis under load changes. Shijin and Udayakumar [8] have introduced a PID controller to adjust the speed of wheeled mobile robots using dynamic and kinematic modeling. Their model considers the errors that emerge between the controller output and the actual speed of the robot. Some other works [9,10,11,12,13] have used velocity control as a partial element of the navigation and obstacle avoidance behavior of mobile robots.

Among intelligent and knowledge-based techniques, fuzzy decision-making plays a vital role in robot navigation with imprecise, incomplete, and vague sensor measurements [14,15]. Besides fuzzy decision-making, machine learning is a powerful technique to make appropriate decisions in the absence of sufficient knowledge. Supervised machine learning (SML) uses a training set to adjust the decision process based on a pre-defined pattern. It can be integrated into fuzzy decision-making to learn based on phenomena data gathered from the environment and can iteratively modify parameters of the fuzzy membership, or can be applied along with a fuzzy controller to make optimal or near-optimal decisions regarding the dynamicity of the environment.

Existing works for the speed control of mobile robots typically do not use intelligent methods, and those that do use intelligent methods usually apply only one intelligent strategy for this process. In contrast, this paper presents an intelligent technique for the speed control of a wheeled mobile robot using a combination of fuzzy decision-making and SML. The technique is aimed to appropriately adjust the speed of a follower mobile robot in a two-robot leader-follower formation. The follower robot is equipped with two ultrasonic sensors installed in its front to constantly measure its distance to the leader robot. The use of two ultrasonic sensors can enhance the accuracy and reliability of the measurements. The follower’s speed is adjusted via the fuzzy controller and the SML algorithm employed in the proposed technique. The fuzzy controller uses both ultrasonic sensory measurements to determine the ultimate distance of the follower to the leader. The sensory measurements often are imprecise due to sensor errors. Fuzzy decision-making can appropriately deal with the imprecise measurements. The SML algorithm specifies a proper speed for the DC motors of the follower robot using the ultimate distance from the fuzzy controller. The advantage of combining a fuzzy controller and the SML algorithm in one technique is that the intelligent inference process is efficient under various phenomena data. Simulation results have shown that the proposed technique preserves the stability of the robot while preventing collisions with the leader. The main contributions of the technique can be highlighted as below:

- Applying two ultrasonic sensors to enhance the accuracy of the distance measurements
- Determination of the ultimate distance with the fuzzy controller based on the measurements from both ultrasonic sensors
- Specifying the speed of the (follower) robot with the SML algorithm based on the ultimate distance

The remainder of the paper is organized as follows. Section 2 presents some background on fuzzy decision-making and machine learning. Section 3 describes the components of the proposed technique in detail, including the fuzzy controller and SML algorithm. Section 4 contains the evaluation results of the proposed technique under different scenarios. Finally, our conclusions are presented in Section 5.

## 2. Background

This section presents a brief description of the main elements and procedures that are used in fuzzy decision-making and supervised machine learning (SML).

**Figure 1:**
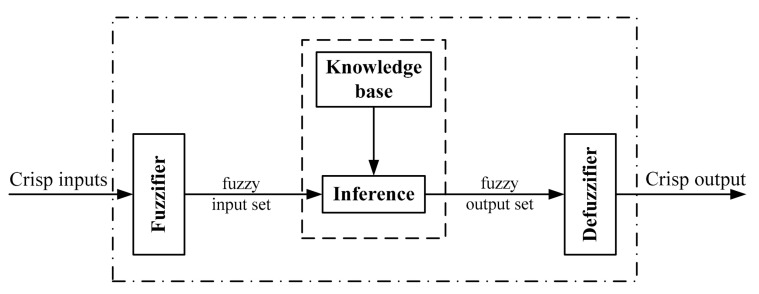
A schematic of fuzzy inference system.

Fuzzy logic [16,17] is a powerful tool to deal with imprecise, vague, and incomplete information in complex systems such as robotic systems. It is based on relative graded membership to resemble human perception and cognition in decision-making in the absence of precise knowledge. All functions of fuzzy decision-making are provided by a fuzzy inference system. As illustrated in Figure 1, this system consists of four main components: fuzzifier, rules, inference engine, and defuzzifier. The fuzzifier unit converts a crisp set of input values to a fuzzy set using membership functions. The fuzzy set of the output is calculated by fuzzy rules through a fuzzy inference process. Finally, the defuzzifier unit converts the output fuzzy set to a crisp value using defuzzification.

**Figure 2:**
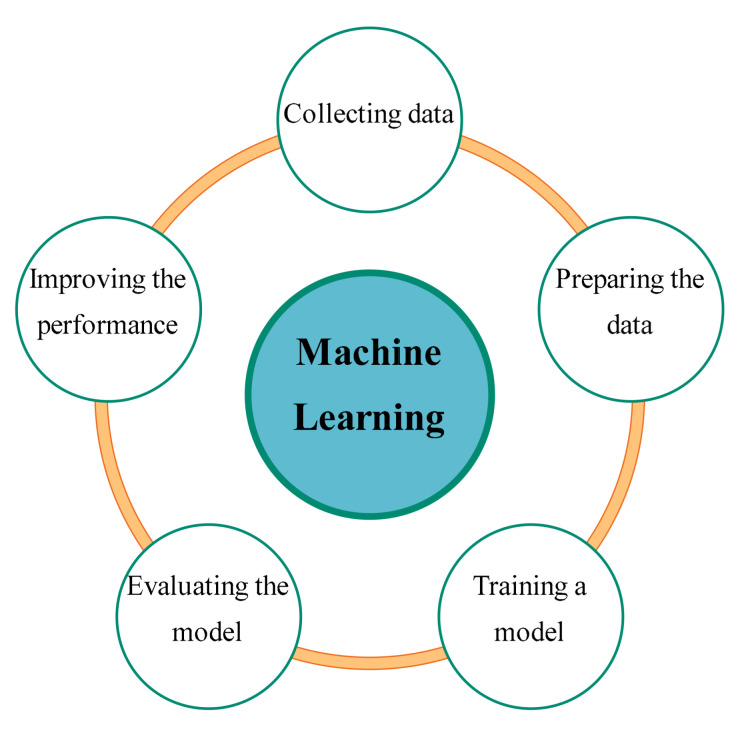
Main steps of the machine learning algorithms.

Machine learning [18] is a subset of artificial intelligence in which computers can learn without being explicitly programmed. It is related to the development process of computer systems in which they can make intelligent decisions in response to new data based on previously gained experience. Figure 2 shows the main steps in machine learning systems. Collecting raw data is the first step of a machine learning system. The data preparation phase determines the quality of data applied in analytical processes. An appropriate learning model is selected and trained in the next phase. The evaluation phase determines the performance of the selected learning model. Finally, the feedback from the evaluation phase is used to improve the model efficiency or replace it with another model. SML [19,20] is one of the machine learning algorithms in which the learning model is trained using a pre-defined training set to improve the accuracy of the decision-making. The linear regression model is a fundamental SML algorithm with two parameters that can be represented as,

(1) $$S\left(x\right)=\alpha +\beta x$$

where α and β indicate parameters that are determined during supervised training of the model. The main goal of the linear regression model in (1) is to predict the best output for each input x. It is worth noting that this paper considers an SML algorithm based on linear regression.

## 3. The Proposed Technique

**Figure 3:**
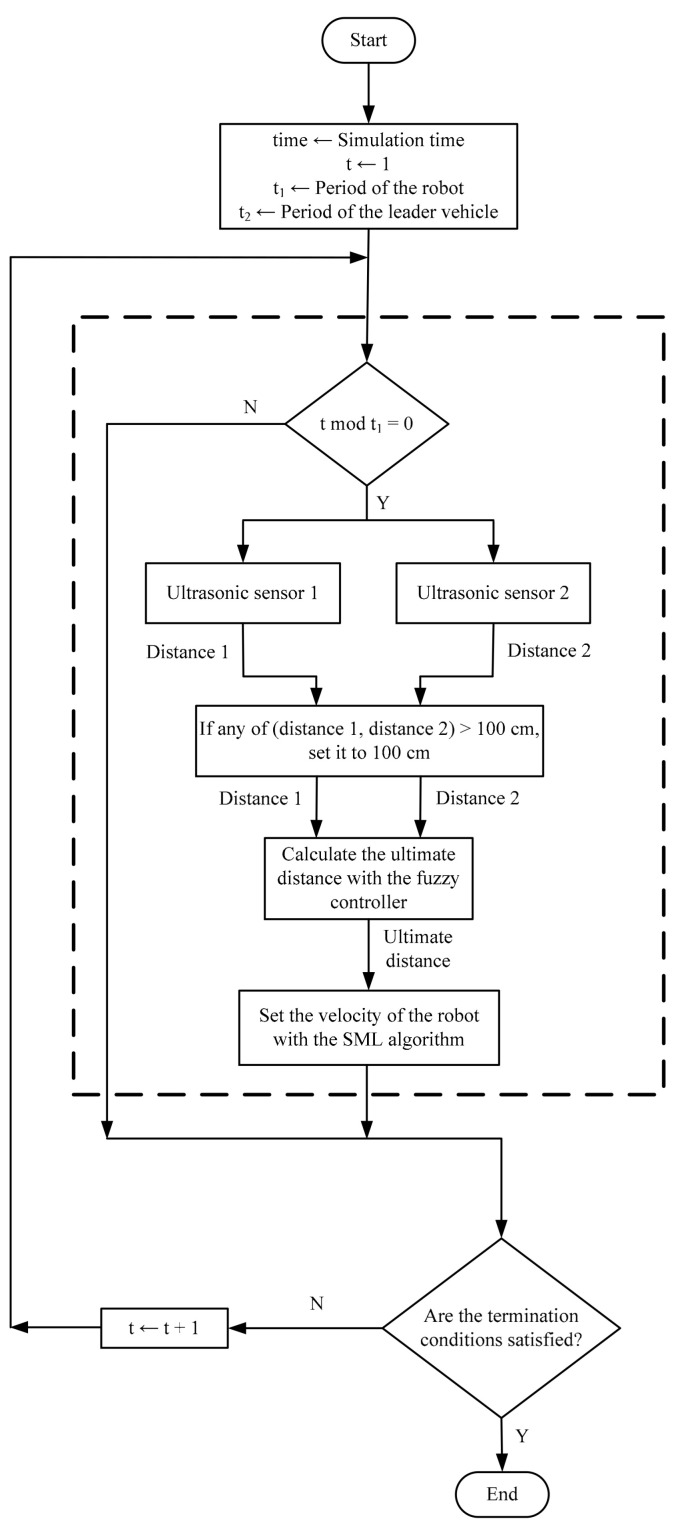
The workflow of the proposed technique for the speed control.

Assume a two-robot leader-follower formation control scenario, where the follower robot steers to maintain a formation with the leader robot. Two ultrasonic sensors, installed on the forward motion direction of the follower robot, continuously measure its distance to the leader robot. Figure 3 shows the workflow of the proposed technique for the speed control of the follower robot. The technique consists of a fuzzy controller and an SML algorithm to specify the ultimate distance to the leader and the speed of the follower, respectively, in order to maintain the formation with the leader robot. The leader robot changes its speed every $t_{2}$ seconds to a random value that is unknown to the follower and subsequently the follower robot adjusts its speed every $t_{1}$ seconds.

### 3.1. The Robot Model

**Figure 4:**
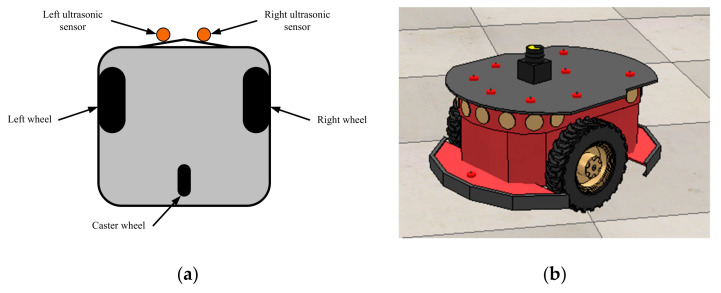
Robot models for theoretical and experimental studies. (**a**) Schematic of a differentially driven mobile robot (the bottom view), (**b**) A V-REP implemented Pioneer 3-DX robot.

Differentially driven mobile robots are very common robot models used for theoretical and experimental studies. The specific mobile robot selected for the proposed speed controller technique implementation was the Pioneer 3-DX [21] (Figure 4). This robot model was employed for the experimental studies in the V-REP robot simulator discussed in Section 4. We have used only two sonars installed in the front of the robot to detect obstacles (here, to detect the leader robot).

### 3.2. The Fuzzy Controller

The fuzzy controller determines the ultimate distance between the follower robot and the leader vehicle based on data readings of the ultrasonic sensors. Beforehand, it should be checked that at least one of the readings of the two sensors is less than or equal to 100 cm. The other measurement, if greater than that threshold, is set to 100 cm. In case both measurements are greater than that threshold, both are set to 100 cm. The rationale behind this is that when these measurements are set to the specified threshold, the speed controller will steer the follower with the maximum speed until the distance to the leader becomes less than 100 cm. Then, the speed controller can adjust the speed appropriately in order to avoid collision with the leader. Note that we assume the follower’s maximum speed is larger than the leader’s actual speed.

**Figure 5:**
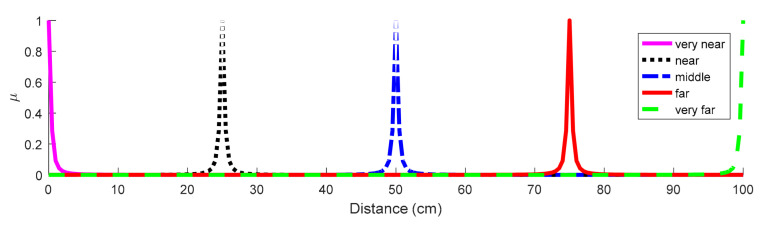
Membership graphs of the fuzzy controller.

The fuzzy controller consists of two input parameters, namely ‘Distance 1’ and ‘Distance 2’, and one output parameter, namely ‘ultimate distance’. The linguistic terms of all the parameters are specified as {very near, near, middle, far, very far}, and their universe of discourse is determined as {0, 1, …, 100} cm. Since the follower navigates in the highest velocity when the distance between the leader and the follower is equal or greater than 100 cm, we considered that it has the maximum value in the universe of discourse. That is, the distances greater than 100 cm will be considered equal to 100 cm. Membership degrees of all the linguistic terms and quantitative amounts are determined by the bell-shaped membership function [22] as follows:

(2) $$F\left(x;a,\;b,\;c\right)=\frac{1}{1+{\left|\frac{x-c}{a}\right|}^{2b}}$$

where x is a member of the universe of discourse, c and a are used to adjust the center and width of the membership function, and b is the slope at the cross points. Figure 5 shows the membership graphs specified for this controller.

Table 1 represents the IF-THEN rules that are used in the fuzzy controller. These rules are designed based on our experiences in a case study, in which Distance 1 and Distance 2 were fed into the fuzzy controller to obtain the safe ultimate distance. However, some of the rules were adopted with human perceptions to enhance the performance of the fuzzy controller. These rules are applied to generate the total rule. The outputs are determined in a way that they can determine an appropriate ultimate distance based on Distance 1 and Distance 2. The fuzzy rules of this controller are specified by the fuzzification unit as below:

Rule 1: If x is ‘very near’ and y is ‘very near’ then $f_{1}$ is ‘very near’
Rule 2: If x is ‘very near’ and y is ‘near’ then $f_{2}$ is ‘very near’
…
Rule 25: If x is ‘very far’ and y is ‘very far’ then $f_{25}$ is ‘very far’

After all of the rules are separately built by the max-min function, they are aggregated together by the max function to produce the total rule as follows:

$${ [\begin{array}{ccc} 1.0000000000 & \ldots & 0.0000099999\\ \ldots & \ldots & \ldots \\ 0.0000099999 & \ldots & 0.0000099999 \end{array}]}_{201\times 201}\wedge { [\begin{array}{ccc} 0.0001599700 & \ldots & 0.0000099999\\ \ldots & \ldots & \ldots \\ 0.0000099999 & \ldots & 0.0000099999 \end{array}]}_{201\times 201}\wedge \ldots \;\wedge { [\begin{array}{ccc} 0.0000099999 & \ldots & 0.0000099999\\ \ldots & \ldots & \ldots \\ 0.0000099999 & \ldots & 1.0000000000 \end{array}]}_{201\times 201}={ [\begin{array}{ccc} 1.000000000 & \ldots & 0.000039998\\ \ldots & \ldots & \ldots \\ 0.000039998 & \ldots & 1.000000000 \end{array}]}_{201\times 201}$$

The total rule is applied to calculate the distance between the robot and the leader vehicle. Algorithm 1 represents an overall view of this procedure. At first, Distance 1 and Distance 2 are separately fuzzified by the fuzzification unit. Then, the input fuzzy set is determined by the AND operation between the fuzzy sets of the distances. The inference process is performed by the Mamdani fuzzy model, as below:

(3) $$\mu_{z}\left(C\right)=\max\; [\min\; [\mu_{x}\left(A\right),\mu_{y}\left(B\right)]]$$

where A and B are the input sets, C is the output set, $\mu_{x}\left(A\right)$ is the membership degree of x in set A, $\mu_{y}\left(B\right)$ is the membership degree of y in set B, and $\mu_{z}\left(C\right)\;$ is the membership degree of z in set C. This process specifies the fuzzy set of the ultimate distance based on the input fuzzy set and the total rule. Finally, the crisp value of the ultimate distance is computed by the defuzzification unit using the center of gravity method [23] as follows:

(4) $$G=\frac{\sum_{i=1}^{n}\mu_{U}\left(x_{i}\right)x_{i}}{\sum_{i=1}^{n}\mu_{U}\left(x_{i}\right)}$$

where U is the universe of discourse, $x_{i}$ is element i th of the fuzzy set U, $\mu_{U}\left(x_{i}\right)$ is the membership degree of $x_{i}$, and n is the number of elements.
**Algorithm 1** Determination of the ultimate distance with the fuzzy controller1LD ← Distance 12RD ← Distance 23D ← Ultimate distance4R ← Total rule5LD_F_ ← ***FUZZIFICATION*** (LD)6RD_F_ ← ***FUZZIFICATION*** (RD)7Input_F_ ← ***AND*** (LD_F_, RD_F_)8D_F_ ← ***INFERENCE*** (Input_F_, R)9D ← ***DEFUZZIFICATION*** (D_F_)10**Return** D

### 3.3. The SML Algorithm

The speed of the robot’s DC motors should be regulated to control the robot’s speed based on the ultimate distance determined by the fuzzy controller. The speed should be determined in a way that the stability of the robot’s speed and the distance between the vehicles will be high. If the distance is short then the speed will be decreased; otherwise, it will be increased accordingly. The SML algorithm specifies the speed of both motors based on the distance between the robot and the leader vehicle.

Table 2 presents some data in the training set that is determined in the controller to find all of the possible solutions to regulate the speed of both DC motors. The distance is in the range of [0, 100] cm, and the speed is in the range of [0, 255] PWM which later is converted to an actual value as m/s. The main algorithm to determine the best solution can be given by linear regression, as below:

(5) $$Diff_{i}=\frac{\sum_{j=1}^{S}\left|\left(\alpha D_{j}+\beta \right)-S_{j}\right|}{S}\;,\forall i\in \left\{1,\ldots ,N\right\}$$

where N is the number of feasible solutions, $Diff_{i}$ is the difference (from the target) for each solution, S indicates the number of selections for each solution, $D_{j}$ represents the distance for each selection, $S_{j}$ is the number of randomized times for each selection, and α, β represent the weighting parameters (to be generated randomly). The weights having the least difference are selected as the best solution.

**Figure 6:**
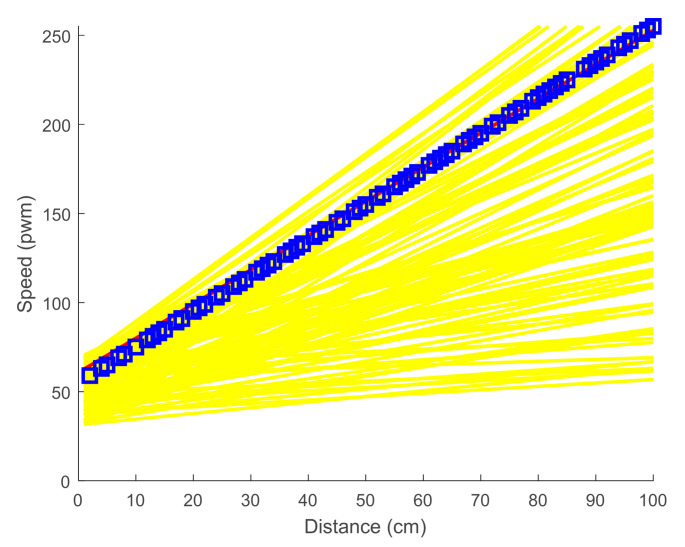
All solutions (yellow lines) and the best solution (red line) of the SML algorithm. Data are demonstrated with blue squares.

All possible solutions are generated after the simulation process based on the training set. Table 3 contains some of the generated solutions. The results are increasingly sorted based on the difference parameter. Therefore, the first solution is selected as the best solution. Figure 6 shows all solutions and the best solution resulting from the simulation process. The blue squares indicate the instances of the training set, the yellow lines indicate all the solutions, and the red line indicates the best solution.

For N=20 randomly generated α and β, the best solution with the minimum difference value $Diff_{i}=0.11188$ is found at α=1.9958 and β=55.265. Therefore, the speed can be predicted by:

(6) Velocity=α×dis+β=1.9958×dis+55.265=2×dis+56

where dis indicates the ultimate distance determined by the fuzzy controller.

Algorithm 2 describes how to find all possible solutions and the best solution for the controller. The values of M, N, P, min_α_, max_α_, min_β_, and max_β_ are considered to equal 75, 100, 50, 0.1, 2.5, 30, and 70, respectively.
**Algorithm 2** Selection algorithm of the SML algorithm**Input:** T ← Training set M ← number of instances in training setN ← number of solutions P ← number of selections for each solution min_α_ ← minimum α; max_α_ ← maximum α min_β_ ← minimum β; max_β_ ← maximum β**Output:** Solutions (S), α, β1begin2 ** for** all N3 ** begin**4   α = (max_α_ − min_α_) × rand() + min_α_5   β = (max_β_ − min_β_) × rand() + min_β_6   sum = 07   **for** all P8   **begin**9    R = round(rand() × M);10     **if** R = 0 **then**11       R = 112     new = α × T(R,1) + β13     sum = sum + abs(new − T(R,2))14  **end;**
15  difference = sum/P16  S(i,1) = α17  S(i,2) = β18  S(i,3) = difference19 
**end;**
20  Sort S increasingly based on the difference values21  α = S(1,1);22  β = S(1,2);23**end**;

## 4. Evaluations

**Figure 7:**
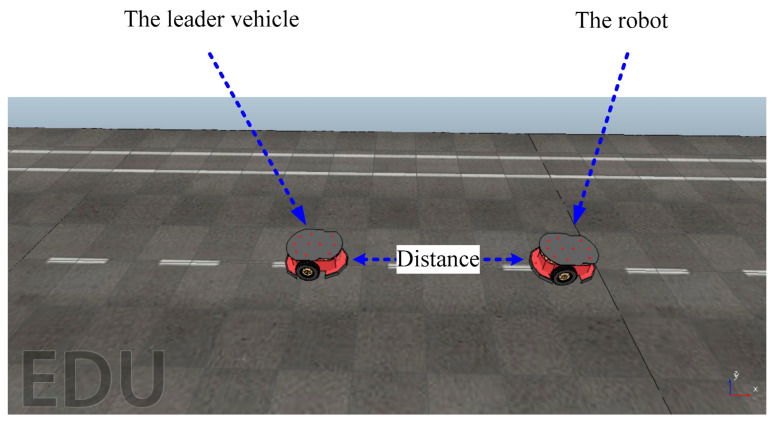
A schematic of the case study using two Pioneer 3-DX robots in V-REP.

The performance of the proposed technique for the speed control was evaluated under different conditions based on a case study that used the Pioneer 3-DX [24,25] to navigate two robots on a road between two positions. Figure 7 shows a schematic of the simulation scenario. The length, width, and height of the robot were 485 mm, 381 mm, and 217 mm, respectively. We used scalability around 70% of the real robot in the simulations. The simulation process was performed using an integration of MATLAB and V-REP in which the model was considered in the V-REP simulator and the robot’s speed was regulated in the MATLAB environment. The robot’s speed was determined in the periodic time t_1_ while the leader vehicle’s speed was specified in the periodic time t_2_. The default values of t_1_ and t_2_ were considered as 2 s and 5 s, respectively. Note that max indicates the maximum speed of the leader vehicle.

**Figure 8:**
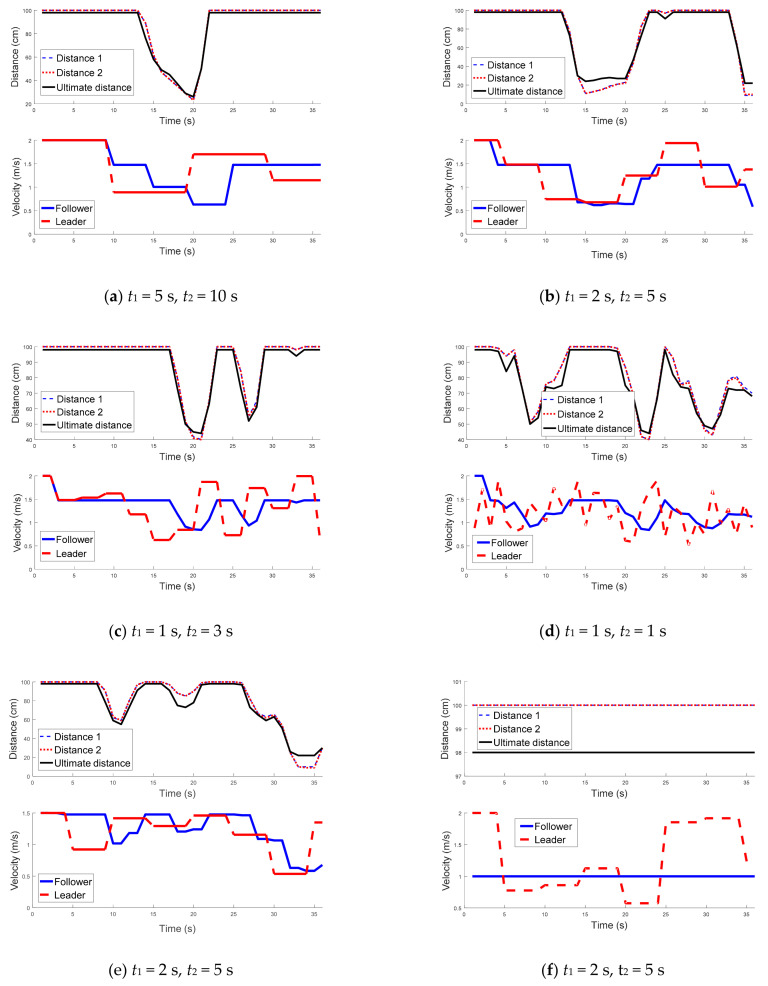
Time histories of the relative distances as well as velocities.

Figure 8 shows the performance of the proposed technique under various assumptions of time parameters t_1_ and t_2_ as well as velocities of the leader. As can be seen in the results, when the leader changed its speed to a random value, the follower’s velocity controller could regulate the follower’s velocity properly, so, the formation was maintained without collision between the two robots. Furthermore, the time histories of the follower’s velocities showed relative smoothness that provided for stable motion. Figure 8f illustrates that when the follower’s speed was constrained to be less than 1 m/s, the controller steered the robot with the maximum speed to keep the formation.

**Figure 9:**
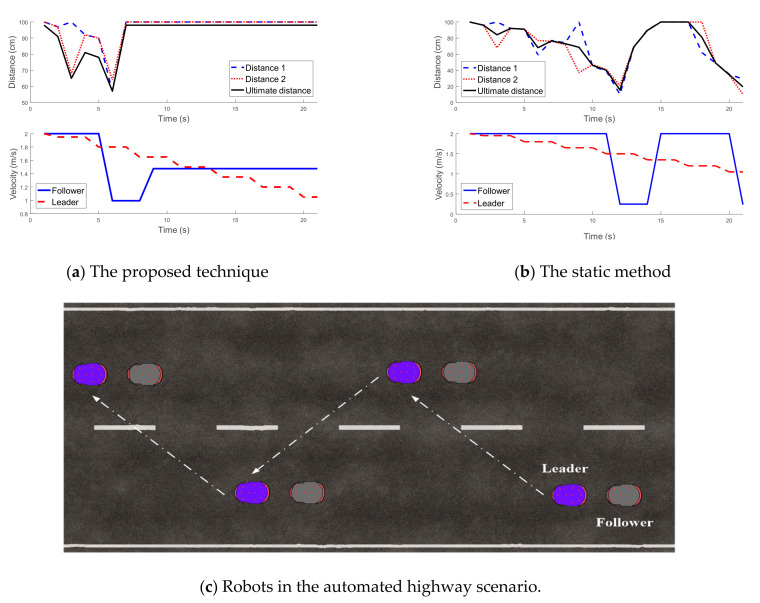
Comparison of results of the proposed technique and the static method.

To compare the proposed technique with a static method under an automated highway scenario, the robots were considered to move down a two-lane road (Figure 9). The leader robot decreased its speed from 2 to 0.5 m/s gradually while in the static method the follower robot adjusted its speed as

(7) $$Speed=\left\{\begin{array}{l} \frac{\left|dis-100\right|}{T},\;\;dis<50\\ \;\;\;2,\;\;\;else \end{array}\right.$$

where dis indicates the average of Distance 1 and Distance 2, and T represents the time interval between estimation steps. The simulation results in Figure 9 demonstrate that when using the static method, the follower could not efficiently adjust its speed to follow the leader with maintaining a regular distance. In contrast, when using the proposed technique, the follower adjusted its speed intelligently and maintained its distance to the leader properly after 7 s.

The simulation results of the proposed technique shown in Figure 8 and Figure 9 demonstrate that the proposed fuzzy controller for determining the distance between the vehicles and the SML algorithm for calculating the robot’s speed worked properly under different conditions. The evidence can be seen in the results, which show that the distance between the vehicles remained in a safe range to avoid any conflicts or collisions between them.

## 5. Conclusions

In wheeled mobile robots, speed control is a very challenging and time-consuming task that is required to appropriately perform the navigation and path-tracking processes. This paper presented an intelligent speed control for a wheeled mobile robot using a fuzzy system and supervised machine learning (SML). Two ultrasonic sensors are applied in front of the robot to measure the distance between the robot and the leader vehicle separately. The use of two sensors can enhance the precision of the distance estimation. Initially, the ultimate distance of the robot from the leader vehicle is specified by the fuzzy controller based on distance measurements from the sensors. Afterward, the SML algorithm computes the speed of the DC motor using the ultimate distance.

The Pioneer 3-DX was applied as a case study in the V-REP simulator through integration with the MATLAB environment to navigate two robots between two positions on a road. Evaluation results indicated that the proposed technique adjusts the speed of the robot properly so that the distance of the robot from the leader vehicle is optimal or near-optimal, and the robot does not collide with the front vehicle. In future work, we will use various types of sensors to improve the precision of the measured distance and speed of the robot.

In future works, the fuzzy controller’s performance will be analyzed under a scenario that assumes one of the sensors has malfunctioned. Additionally, various techniques (e.g., neural networks) will be incorporated into the controller to increase the learning capability of the system.

## Figures and Tables

**Table 1:** IF-THEN rules of the fuzzy controller to determine the ultimate distance.

Rule No.	Inputs		Output
	Distance 1	Distance 2	Ultimate Distance
1	very near	very near	very near
2	very near	near	very near
3	very near	middle	near
4	very near	far	near
5	very near	very far	near
6	near	very near	very near
7	near	near	near
8	near	middle	near
9	near	far	middle
10	near	very far	middle
11	middle	very near	near
12	middle	near	near
13	middle	middle	middle
14	middle	far	middle
15	middle	very far	middle
16	far	very near	near
17	far	near	middle
18	far	middle	middle
19	far	far	far
20	far	very far	far
21	very far	very near	near
22	very far	near	middle
23	very far	middle	middle
24	very far	far	far
25	very far	very far	very far

**Table 2:** Some data in the training set of the SML algorithm.

#	Distance (cm)	Speed (pwm)
1	28	111
2	46	147
3	90	235
4	14	83
5	2	59
6	37	129
7	57	169
8	77	209
9	100	255
10	83	221
11	70	195
12	53	161
13	33	121
14	10	75
15	4	63
16	36	127
17	58	171
18	76	207
19	94	243
20	98	251

**Table 3:** Some of the solutions of the SML algorithm.

#	α	β	Difference
1	1.9958	55.265	0.11188
2	2.0416	53.442	1.1689
3	2.0207	58.254	4.3476
4	2.1844	43.425	4.5341
5	2.1849	43.375	4.7434
6	1.8035	61.932	4.8971
7	2.1776	49.905	5.3224
8	1.8547	67.367	5.4634
9	2.1408	42.899	5.547
10	1.8535	57.073	5.5498
11	1.7426	68.478	6.0083
12	2.2565	37.729	6.158
13	2.2412	39.78	6.2725
14	2.1326	41.218	6.3085
15	2.3216	33.757	8.5793
16	2.3812	40.222	9.4291
17	1.917	68.832	9.6143
18	2.2925	32.29	9.664
19	2.3279	47.099	9.9256
20	2.2919	46.491	10.077

## Data Availability

Not applicable.

## References

[1] Da Mota F.A.X., Rocha M.X., Rodrigues J.J.P.C., De Albuquerque V.H.C., De Alexandria A.R. (2018). Localization and navigation for autonomous mobile robots using petri nets in indoor environments. IEEE Access.

[2] Gonzalez A.G.C., Alves M.V.S., Viana G.S., Carvalho L.K., Basilio J.C. (2017). Supervisory control-based navigation architecture: A new framework for autonomous robots in industry 4.0 environments. IEEE Trans. Ind. Inform..

[3] Gharajeh M.S., Jond H.B. (2020). Hybrid Global Positioning System-Adaptive Neuro-Fuzzy Inference System based autonomous mobile robot navigation. Robot. Auton. Syst..

[4] Al Khatib E.I., Jaradat M.A.K., Abdel-Hafez M.F. (2020). Low-Cost Reduced Navigation System for Mobile Robot in Indoor/Outdoor Environments. IEEE Access.

[5] Dirik M., Kocamaz A.F., Castillo O. (2020). Global Path Planning and Path-Following for Wheeled Mobile Robot Using a Novel Control Structure Based on a Vision Sensor. Int. J. Fuzzy Syst..

[6] Kodagoda K.R.S., Wijesoma W.S., Teoh E.K. (2002). Fuzzy Speed and Steering Control of an AGV. IEEE Trans. Control Syst. Technol..

[7] Dursun E.H., Durdu A. (2016). Speed control of a DC motor with variable load using sliding mode control. Int. J. Comput. Electr. Eng..

[8] Shijin C.S., Udayakumar K. Speed control of wheeled mobile robots using PID with dynamic and kinematic modelling. Proceedings of the International Conference on Innovations in Information, Embedded and Communication Systems (ICIIECS).

[9] Algabri M., Mathkour H., Ramdane H., Alsulaiman M. (2015). Comparative study of soft computing techniques for mobile robot navigation in an unknown environment. Comput. Hum. Behav..

[10] Sadrfaridpour B., Saeidi H., Burke J., Madathil K., Wang Y. (2016). Modeling and control of trust in human-robot collaborative manufacturing. Robust Intelligence and Trust in Autonomous Systems.

[11] Nasrinahar A., Chuah J.H. (2018). Intelligent motion planning of a mobile robot with dynamic obstacle avoidance. J. Veh. Routing Algorithms.

[12] Mohanta J.C., Keshari A. (2019). A knowledge based fuzzy-probabilistic roadmap method for mobile robot navigation. Appl. Soft Comput..

[13] Aouf A., Boussaid L., Sakly A. (2019). Same fuzzy logic controller for two-wheeled mobile robot navigation in strange environments. J. Robot..

[14] Qureshi M.S., Swarnkar P., Gupta S. (2018). A supervisory on-line tuned fuzzy logic based sliding mode control for robotics: An application to surgical robots. Rob. Auton. Syst..

[15] Xiang X., Yu C., Lapierre L., Zhang J., Zhang Q. (2018). Survey on fuzzy-logic-based guidance and control of marine surface vehicles and underwater vehicles. Int. J. Fuzzy Syst..

[16] Khan S.A., Daachi B., Djouani K. (2012). Application of fuzzy inference systems to detection of faults in wireless sensor networks. Neurocomputing.

[17] Ferdaus M.M., Anavatti S.G., Pratama M., Garratt M.A. (2020). Towards the use of fuzzy logic systems in rotary wing unmanned aerial vehicle: A review. Artif. Intell. Rev..

[18] Michalski R.S., Carbonell J.G., Mitchell T.M. (2013). Machine Learning: An Artificial Intelligence Approach.

[19] Kotsiantis S.B. (2007). Supervised Machine Learning: A Review of Classification Techniques. Informatica.

[20] Jiang T., Gradus J.L., Rosellini A.J. (2020). Supervised machine learning: A brief primer. Behav. Ther..

[21] Pioneer 3-DX. http://www.generationrobots.com/.

[22] Mamdani E.H., Assilian S. (1975). An experiment in linguistic synthesis with a fuzzy logic controller. Int. J. Man Mach. Stud..

[23] Dubois D., Prade H. (2012). Fundamentals of Fuzzy Sets.

[24] Patelis D. (2012). Commissioning of the Pioneer Robot. Bachelor’s Thesis.

[25] Sharma V. (2018). Pioneer Robot. Master’s Thesis.

